# Integration of smart glasses in telementoring for simulated medical procedures

**DOI:** 10.1590/acb403625

**Published:** 2025-05-09

**Authors:** Tiago Francisco Meleiro Zubiolo, Vlaudimir Dias Marques, Miyoko Massago, Aline Cardoso Machado, Felipe Hideaki Ueda, Mateus de Amorim Aboboreira, Eduardo Filgueiras Damasceno, Sanderland José Tavares Gurgel, Carlos Edmundo Rodrigues Fontes, Luciano de Andrade

**Affiliations:** 1 Universidade Estadual de Maringá – Postgraduate Program in Management, Technology and Innovation in Urgency and Emergency – Maringá (PR) – Brazil.; 2 Universidade Estadual de Maringá – Maringá (PR) – Brazil.; 3 Universidade Estadual de Maringá – Postgraduate Program in Health – Maringá (PR) – Brazil.; 4 Universidade Tecnológica Federal do Paraná – Academic Department of Computing – Cornélio Procópio (PR) – Brazil.; 5 Universidade Estadual de Maringá – Department of Medicine – Maringá (PR) – Brazil.

**Keywords:** Telemedicine, Smart Glasses, Simulation Training, Augmented Reality

## Abstract

**Purpose:**

To demonstrate the complete process of telementoring implementation using augmented-reality smart glasses for performing a simulated medical procedure.

**Methods:**

Fifteen participants, including physicians and medical students, were recruited to participate in a telementoring session with an educational focus during simulated thoracostomy with closed pleural drainage. A questionnaire assessing technology usability and usefulness was administered, and the results were analyzed by Cronbach’s alfa and multiple correspondence analysis (MCA).

**Results:**

The mean age of the participants was 28.8 and 66.67% of them were women. The test results indicated a Cronbach’s alpha of 78% and an MCA of 49.48% for the first three dimensions.

**Conclusion:**

The results showed that smart glasses are easy to use and facilitate communication among the professionals involved, providing comfort and safety to participants of care simulations. Additionally, smart glasses were considered to have perceived usefulness.

## Introduction

Improving access to healthcare is a global challenge, especially in underdeveloped and developing countries. An increased incidence of deaths has been reported among critically ill patients treated in remote and/or rural emergency units compared with urban settings^1^.

In Brazil, approximately 70% of the population does not have supplemental healthcare insurance^2^. According to the 2019 National Health Survey conducted by the Brazilian Institute of Geography and Statistics, public emergency and urgent care units are the first choice of care for an estimated 14.1% of the population^2^. This behavior generates a demand overload for emergency services, further increasing the challenges faced by these institutions^2^. To minimize this problem in Brazil and worldwide, the World Health Organization developed in 2021 the Global Strategy on Digital Health 2020–2025, emphasizing the need to prioritize investments in digital technologies for medical care^3^.

With the improvement in communication technologies and the widespread adoption of the internet and electronic devices, telemedicine has gained prominence worldwide^3^. Additionally, the COVID-19 pandemic and the social distancing measures imposed in response to it have contributed to accelerating the development of telemedical solutions^4^. However, numerous issues still hinder the adoption of telemedical technologies, such as a lack of specialists present at the procedure site, low acceptance rates, inadequate services, precarious professional resources, lack of materials and equipment, poor mobile phone and internet coverage, among others^3^.

An interesting technology that has emerged in this context is telementoring. It allows specialists to provide remote guidance, which is particularly important in isolated areas or small- and medium-sized hospitals, where the presence of a specialist physician is often limited^5^. In a comprehensive review of the literature on the use of telementoring for the care of burn and trauma patients, Lapointe et al.^6^ reported improvements in diagnosis and decreased transfer and hospitalization times in the studied population^7^.

Among various innovative technologies, smart glasses equipped with augmented reality have garnered increasing attention across several fields^6^. Augmented reality has great potential to provide powerful, contextual, and *in loco* learning experiences^8^. It can be used to perform a wide variety of tasks, including displaying and manipulating information in the field of view of users, mapping virtual images to real objects, and video conferencing^9^.

A scoping review was conducted by Munzer et al.^10^ with the aim of evaluating the use of augmented-reality smart glasses in emergency medicine. The authors found 24 open-access articles published between 2005 and 2019; 12 focused on education and training, and the other 12 examined topics directly related to patient care, including triage care in natural disasters and mass events, cardiopulmonary resuscitation, insertion of central venous access devices, and tumor resection planning.

Although novel technologies are widely adopted and discussed in the context of medicine, to the best of our knowledge, no nationwide study has used augmented-reality smart glasses in real or simulated care environments. To contribute to the theme, we aimed to demonstrate the feasibility of integrating augmented-reality smart glasses in the telementoring of thoracostomy with closed pleural drainage in a simulated environment.

## Methods

### Study design, location, and participants

This is a proof-of-concept feasibility study of a technological solution. The study was conducted from April to June 2023 at Universidade Estadual de Maringá, Paraná state, Brazil. The experimental protocol was approved by the Research Ethics Committee (protocol No. 58657122.7.0000.0104), ensuring compliance with all necessary ethical standards.

Study participants comprised physicians and 6th-year undergraduate medical students. Participants were selected from a list of professionals and students affiliated with the hospital where the study was conducted and were invited to participate voluntarily outside their work/internship shifts. The inclusion criteria were:

- Being a physician or a final-year medical student;
- Agreeing to participate in the study.

No prior experience with augmented reality (AR) was required. To assess participants’ familiarity with thoracostomy procedures and AR devices, a brief pre-study questionnaire was administered, gathering information on their previous exposure, frequency of performing thoracostomies, and any prior use of AR-related technology in medical training. Additionally, emergency and thoracic surgery specialists were invited to provide remote guidance during the study. All participants, including remote specialists, reviewed and signed an informed consent form before participation.

### Augmented-reality smart glasses for telementoring

**Figure 1:**
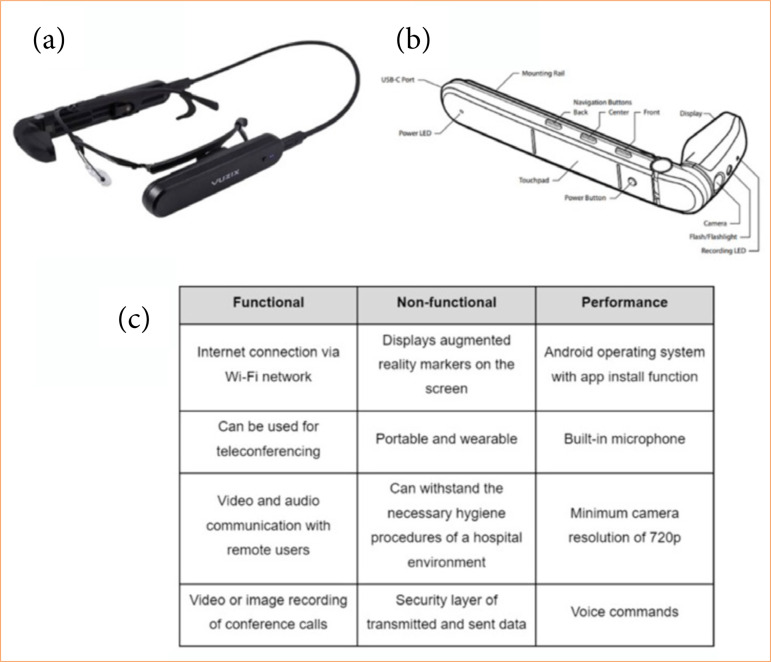
Structure, components, and features of smart glasses for telemonitoring.. **(a)** Vuzix M400 glasses connected to an external battery module. **(b)** Components of the Vuzix M400 glasses, including a display, camera, touchpad, navigation buttons (back, center, and front), USB-C port, power LED, recording LED, and a mounting rail for accessories. **(c)** Smart glasses requirements and specifications.

Source: Elaborated by the authors.

The wearable device used in this study was a pair of M400 smart glasses (Vuzix Technologies, Rochester, New York, United States of america) (Fig. 1a). Developed to be used with voice and gesture commands, the device features an autofocus camera, built-in microphone and speaker, and expansive OLED monocular display, enabling real-time visualization of the procedure environment (Fig. 1b). The Smart Glass Requirements and Specifications of the smart glasses are shown in Fig. 1c. Smart glasses meeting these requirements were deemed suitable for use in an educational simulated procedure based on augmented reality and videoconferencing technologies.

Telementoring was provided via TeamViewer (TeamViewer Technologies, Göppingen, Germany). This application supports augmented reality in the meeting environment. The smart glasses were connected to a mobile hotspot of the educational institution. Specialists used their own laptops with TeamViewer software, speakers, and microphone for the simulation. The augmented reality software (TeamViewer) associated with smart glasses allowed the healthcare professional to follow the entire procedure in real time through the notebook screen.

TeamViewer offers numerous augmented reality features, such as markers (tridimensional objects highlighting specific areas and objects in the real world), annotations (lines or texts used to provide directions, highlight important areas, or add comments), and communication (chat, voice, and video).

### Description of the simulated procedure

The procedure took place in a medical skills laboratory and consisted of a simulation of an emergency room patient with chest trauma. Emergency room professionals assessed the clinical case and provided information on the patient’s history and physical examination findings to the specialist physician through TeamViewer. On-site professionals performed a primary assessment, identifying a condition of traumatic pleural disease that required drainage. They performed a closed thoracostomy with water-seal drainage, receiving guidance and real-time instructions from the specialist physician by videoconference. All steps in the simulated procedure followed the recommendations of the 10th edition of Advanced Trauma Life Support^11^, with the necessary adaptations for an educational simulated environment.

### Data collection and analysis

For assessment of the usability and usefulness of smart glasses, an online survey was conducted after the simulated procedure. The questionnaire contained 15 multiple-choice questions rated on a 5-point Likert scale regarding prior knowledge, usability, communication, and usefulness of smart glasses (Table 1). The questionnaire was constructed using Google Forms (Google, Mountain View, California, United States of America). Questions were developed based on the principles of the technology acceptance model, proposed by Davis in 1989, namely perceived usefulness and perceived ease of use^12^. The first one reflects the expected impact on performance, while the latter indicates the effort required for use. These factors influence the intention to use, ultimately determining technology adoption^12^.

**Table 1:** Questionnaire for evaluating the usability and usefulness of augmented reality smart glasses based on the technology acceptance model.

Code	Affirmation	Theoretical mastery
Q1	I feel confident to perform the procedure	Prior knowledge/experience
Q2	The glasses were easy to wear	Usability
Q3	The glasses fit my face well	Usability
Q4	The glasses did not hinder my clinical performance	Usability
Q5	The smart screen was easy to see	Usability
Q6	I could hear the specialist well	Communication
Q7	I found easy to communicate with the specialist	Communication
Q8	I found it easy to receive information from the specialist	Communication
Q9	The image is sharp and has good resolution	Usability
Q10	Image mirroring did not interfere with my performance	Usability
Q11	Wearing smart glasses will make my day-to-day work easier	Usefulness
Q12	The use of smart glasses by professionals may benefit patients	Usefulness
Q13	I felt comfortable using voice commands	Usability
Q14	I would like to have this tool available at work	Usefulness
Q15	I can easily adapt my routine to use smart glasses in procedure	Usefulness

Source: Elaborated by the authors.

The Likert scale is commonly used to measure respondents’ attitudes, opinions, and feelings in surveys and questionnaires. It consists of a series of statements or items to which participants must respond by indicating their level of agreement or disagreement on an ordinal scale, usually ranging from “strongly disagree” to “strongly agree”^13^.

Given the categorical nature of response options, in which values do not have a significant numerical distance from each other, the Likert scale cannot be treated as a quantitative variable^14^. Multiple correspondence analysis (MCA) is a suitable statistical approach for categorical variables, such as answers rated on a Likert scale. MCA allows exploring patterns and relationships between response categories, facilitating the representation of such relationships in two- or three-dimensional graphs for improved visual interpretation^14^. The positions of the categories of each variable on the multidimensional plane can be interpreted as associations, referred to as domains. MCA was carried out, and the results were plotted on a three-dimensional graph using the factoextra package of R Studio software.

Reliability analysis was conducted using the Cronbach’s alpha coefficient, with a cutoff set at 70%. Cronbach’s alpha reflects the degree of covariance among the items on a scale: the higher the coefficient, the greater the internal reliability of the scale. A Cronbach’s alpha below 70% indicates low reliability, while a value above 90% may be related to redundancies or duplications.

## Results

A total of 15 participants completed the usability questionnaire after performing the simulated procedure with smart glasses. The mean age of participants was 28.8 ± 4.1 years old, and 66.67% (9/15) of them were women. Four participants were specialists in general surgery, one was a specialist in clinical medicine, five were 6th-year medical students, one was a general practitioner, and four were general surgery residents. All individuals worked in the emergency room of the hospital belonging to the research institution. Among graduated professionals, the mean years of experience in medicine was 3.44 ± 2.19 years and 2.0 ± 1.73 years in the area of interest (including medical residency).

### Evaluation of the usability and usefulness of smart glasses

**Figure 2:**
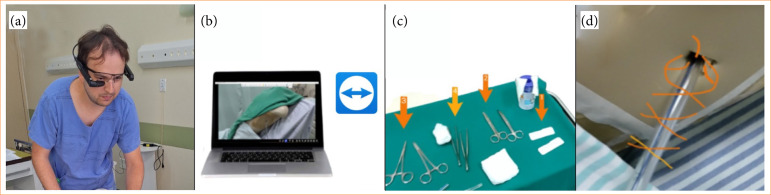
Application of augmented reality in telemonitoring using smart glasses. **(a)** An operator wearing the Vuzix M400 smart glasses, connected to the remote specialist’s view via TeamViewer **(b)** Specialist’s laptop with TeamViewer Assist AR **(c)** Numerical markers added by augmented reality **(d)** Freehand drawing made on a frozen image using augmented reality.

Source: Elaborated by the authors.

Cronbach’s alpha revealed a reliability of 78%, reflecting the participants’ understanding of the usability and usefulness aspects of the smart glasses. Only two questions received negative scores (strongly disagree/disagree), namely Q1 (I feel confident to perform the procedure) and Q13 (I felt comfortable using voice commands). All the other 13 questions were rated positively (Fig. 2).

**Figure 3:**
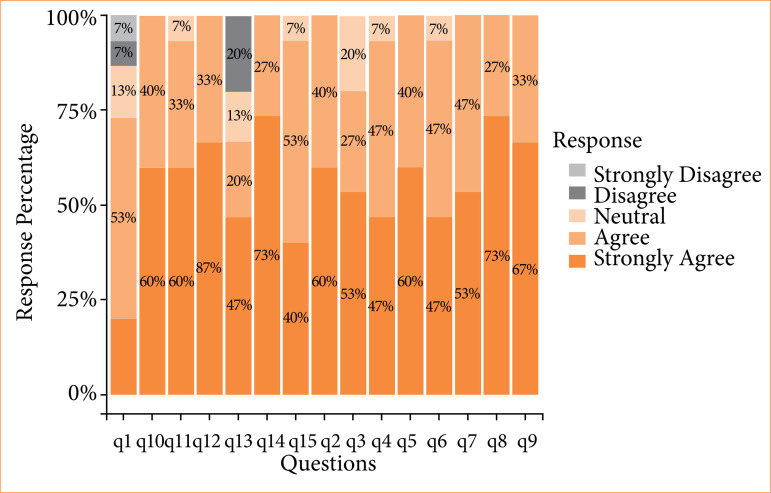
Distribution questionnaire responses assessing the usability and usefulness of smart glasses, rated on a Likert scale.

Source: Elaborated by the authors.

MCA was conducted to gain a better understanding of the results. A three-dimensional graph of the data is shown in Fig. 3. The first dimension was centered exclusively on statements related to usability (Q2, Q5, Q8, Q9, Q11, Q13, and Q14). The second dimension encompassed two statements related to communication (Q6 and Q1) and one related to usability (Q10). The third dimension comprised four statements regarding usefulness (Q4, Q7, Q12, and Q15) and one statement related to usability (Q3). The graph promoted a more comprehensive analysis of emerging patterns.

**Figure 4:**
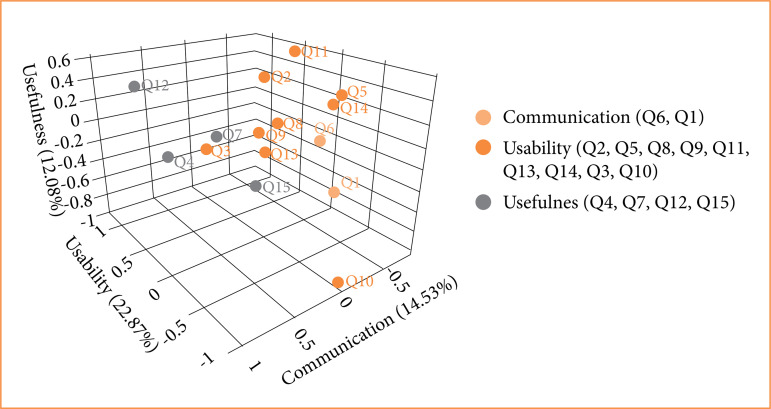
Results of multiple correspondence analysis plotted on a three-dimensional graph.

Source: Elaborated by the authors.

Dimension 1 explained 22.87% of the variance in responses, dimension 2 explained 14.53%, and dimension 3 explained 12.08%. Therefore, the three dimensions of MCA explained 49.48% of the variance in results and exhibited good internal reliability (Fig. 4).

## Discussion

This study simulated a thoracostomy procedure using smart glasses to evaluate their usability and potential application in clinical practice. A questionnaire based on the technology acceptance model was proposed to the participants, and the results demonstrated high internal agreement, both in terms of perceived usability and perceived ease of use. The main assessed dimension, usability, accounted for 22.87% of the total variance, suggesting a promising role for smart glasses in medical settings. These findings support the hypothesis that smart glasses can serve as a telementoring tool for physicians with less experience in performing thoracostomy with closed pleural drainage. Moreover, this study aligns with previous research conducted in the United States of America^15^ and Thailand^16^.

A similar pattern of findings was observed by McTavish et al.^17^, who obtained mainly positive scores in a usefulness/usability test for a simulated environment. However, they also encountered challenges related to internet access quality, with occasional signal outages. These issues highlight the importance of considering technical barriers when evaluating the adoption of new technologies in medical practice.

Although most responses to the questionnaire were positive, there was some negative feedback, particularly concerning the prior knowledge of the procedure. This can be attributed to the inclusion of 6th-year medical students in the sample, who, despite being comfortable with electronic devices, have limited clinical experience.

The relatively young average age of the participants should be also considered when interpreting the results, as this demographic tends to adapt more quickly to new technologies. On the other hand, the average age of physicians in Brazil is 44.9 years old^18^. However, the increasing integration of technology into medical practice suggests that digital tools are being more widely adopted across different age groups. Future studies with a broader age range of participants could help assess whether the findings observed in this younger group extend to more experienced physicians.

This study utilized Vuzix 400 smart glasses. Devices from other brands, such as Google Glass, Microsoft HoloLens, and Epson Moverio, may also find similar applications in healthcare and computing^19^^,^ 20. A prototype study revealed other analyses that highlight more complex requirements for smart glasses, such as durability, minimalist design, ease of use, seamless integration with existing systems, and secure data management^21^. For instance, smart glasses have been increasingly used in medical assistance, supporting injured individuals, computer-human interactions, and image visualization over the years^22^^,^ 23.

Although the results demonstrate the feasibility of using smart glasses in a simulated environment, several challenges must be considered for their broader adoption in real-world clinical settings. High cost remains a significant barrier, particularly in public healthcare systems or resource-constrained environments. Additionally, the scalability of this technology depends on the availability of adequate infrastructure, including stable and secure internet networks, as well as the acceptance of smart glasses by healthcare professionals.

It is important to consider that the issue of internalizing new technologies is addressed, the question regarding infrastructure occurs. Although this is a point to be considered, the use of smart glasses requires a simple infrastructure, and this is a point that indicates the potentiality of using this resource as proposed in this study. Thus, both what refers to infrastructure and organizational support, even if some adjustments are required, do not include something whose complexity could harm the application of the proposal.

Another challenge involves technical limitations, such as ergonomic difficulties associated with prolonged use^22^^,^ 23. A study using a prototype of smart glasses evaluated how the stiffness of the glasses frame affects comfort. They found that increasing the stiffness significantly increases discomfort over prolonged use (60 minutes), especially among women and older participants^24^.

Therefore, while smart glasses hold great potential for revolutionizing remote medical assistance, their successful implementation requires strategies to overcome these barriers and ensure sustainable long-term benefits.

Investment in technologies such as surgical telementoring can be a powerful driver of improved access to healthcare services, especially in remote areas and small- and medium-sized hospitals, where specialist physicians are often unavailable^2^^,^ 3 ^,^ 5. Augmented-reality smart glasses represent one of the most emerging strategies in surgical telementoring worldwide^7^^,^ 8. To the best of our knowledge, no study in Brazil had evaluated the use of augmented-reality smart glasses in real or simulated healthcare procedures yet.

There are some methodological limitations to the current study. The simulation was conducted in a single healthcare center. The sample size was small, and no pre-simulation assessment was made to evaluate participants’ prior knowledge. In addition to the infrastructure and cost issues involved in implementing the technology, there are human factors involved, such as experience with computers, value given to patient care, technology-associated anxiety, structural conditions, and organizational support^25^. In addition, the fact that the sample studied was relatively young (28.8 years old) may limit the generalizability of the study to other populations, due to the familiarity of younger individuals with advances in technology. To mitigate this situation, ongoing education of the team is required to support the adaptation to incorporate new assistance and work technologies.

Given these considerations, future studies must aim to overcome such limitations by including a more extensive and diverse sample and testing the application of augmented reality in real-world situations. Expanding the scope to encompass a variety of medical procedures and spreading the use of these devices are critical steps to enhance the safety, reliability, and usefulness in daily clinical practice. Augmented-reality smart glasses may prove valuable not only for physicians but also for other healthcare professionals.

## Conclusion

The use of augmented-reality smart glasses in a simulated environment showed promise, demonstrating considerable usability and potential benefits for physicians and patient safety. It is expected that smart glasses will be increasingly implemented in healthcare soon, ensuring safety and effectiveness for patients and healthcare professionals.

## Data Availability

The data are available in Figshare at: https://figshare.com/s/a0ebf26c7529fc1e1c4a.
